# Estimation method of attentional object using spatial frequency selectivity in pupillary response

**DOI:** 10.1371/journal.pone.0356996

**Published:** 2026-08-31

**Authors:** Yuhong Lv, Rumi Hisakata, Hirohiko Kaneko

**Affiliations:** Department of Information and Communication Engineering, Institute of Science Tokyo, Tokyo, Japan; Museo Storico della Fisica e Centro Studi e Ricerche Enrico Fermi, ITALY

## Abstract

This study investigates pupillary dynamics associated with object-based visual attention using two overlaid filtered icon images (hybrid stimulus). Subjects were presented with the hybrid stimulus and instructed to maintain their attention on one of the icon images while the spatial frequency (HSF: High spatial frequency or LSF: Low spatial frequency) components of the two images alternated periodically, where they had different components from each other. Pupil diameter was continuously recorded during the alternations. The combination of motion conditions (static or dynamic) of the icon images in the hybrid stimulus was manipulated. Results revealed that pupil size was relatively small when the spatial frequency component of the attentional icon image was HSF and tended to constrict as the spatial frequency component of the attentional icon image changed from LSF to HSF. In contrast, pupil size was relatively large when the spatial frequency component of the attentional icon image was LSF and tended to dilate when the icon image changed from HSF to LSF. These pupil size changes were particularly prominent under the combination of two dynamic or one dynamic and one static icon images. Based on these findings, a method to infer the attended icon image using pupil size change was proposed. The accuracy of the inference method exceeded 70% in specific conditions, suggesting its feasibility as an attention-based information input system for users with limited motor capabilities.

## Introduction

Effective communication is essential for human social interaction and daily life. Humans typically convey information through multiple methods, for example, verbal communication, body language, gesture, or facial expression. Even gaze direction can communicate information, for example by indicating a person’s attention or intention [1–3]. However, for those with severe motor impairments, such as cerebral infarction and Amyotrophic Lateral Sclerosis (ALS), it is difficult to use normal means of communication, such as speech, gesture for human communications and mouse and keyboard for human-computer communications. Therefore, it is needed to develop innovative and user-friendly interface systems for such situations.

In the development of such human-computer interface (HCI) systems, technologies based on non-invasive signals such as eye-movements, electroencephalogram (EEG), electrooculogram (EOG), functional magnetic resonance imaging (fMRI) and near-infrared spectroscopy (NIRS) are used [4–6]. Among these methods, those using EEG, fMRI and NIRS signals are difficult to use for general users because they are costly, challenging to set up for nonspecialists, and require some training. Compared to these, the systems using eye movements signals are relatively easier to use for general users because the devices are simpler and the operation is easier.

Information input systems using eye movement signals have become widely used and are now considered essential communication tools for patients who have lost speech and limb function due to accidents or strokes, as well as for those with severe paralysis mentioned above. For example, text input systems utilizing voluntary eye movements, such as gaze position, fixation duration, blinking, and patterns of eye movement, have become more widespread due to their relative affordability and operational simplicity and are available in the market. People also, in daily life, estimate where other people are paying attention by observing their eye movements. However, for patients in the advanced stages of progressive neurodegenerative conditions such as complete locked-in syndrome (CLIS), who may eventually lose the ability to control eye movements, gaze-based information input systems can become inoperable. Therefore, alternative communication systems that do not depend on voluntary eye movements are needed [7–9]. In addition, information input systems that rely on voluntary eye movements require calibration prior to use in order to establish the correspondence between subjective gaze position and target position.

To develop alternative communication methods that do not rely on voluntary motor control, it is important to identify physiological signals that reflect cognitive or attentional states of humans. Pupil response, an involuntary eye response, has the potential to serve as an alternative input signal for eye movements (changes in gaze position). The pupil primarily reacts to changes in light, a function known as the Pupillary Light Reflex (PLR). Studies have shown that, besides the luminance of the fixating object, pupil size can also vary due to factors such as cognitive load [10], mental effort [11], brightness at the position of attentional focus [12,13], and the imagined brightness [14]. For example, a communication method has been proposed that uses pupillary response to a cognitive load, such as mental arithmetic, in patients with locked-in syndrome [15]. Mathot et al. (2016) proposed an HCI system that utilizes pupil response and covert attention [16]. The subjects attended to one of the two disks positioned on opposite sides of the screen while maintaining their gaze on a central green dot. The brightness of two disks alternated between dark and bright oppositely, causing pupil size to vary depending on the brightness of the attending disk. Specifically, subjects’ pupils dilated when the attentional disk shifted from bright to dark and constricted when it changed from dark to bright. Using this relationship, the system can infer the disk that the subject is covertly paying attention to. However, this approach involves certain considerations: for general users, separating attentional position from gaze position can be demanding; eliciting a detectable pupillary response requires substantial changes in stimulus luminance, and the resulting flicker may reduce viewing comfort. Besides, when two spatially separated icons are presented, it is possible that in patients with severely limited gaze shifts, neural responses may be biased toward the icon that aligns with their existing gaze direction, thereby reducing the system’s ability to detect the response to the truly intended target icon.

In the following experiments, we aimed to find a way to infer an attentional object using hybrid stimulus composed of images containing either HSF or LSF components. Hu et al. (2018) showed that the absolute magnitude of pupil response was different when the spatial frequency components of the attentional image were different [17]. Based on this fact, it is possible to infer the image the subject is paying attention to based on the combination of pupil response and spatial frequency components of the image, therby enabling the development of an attention-based information input system. In our preliminary experiments, however, we found the absolute difference in pupil response when viewing images with different spatial frequency components was relatively small, so that the accuracy for inferring the attentional image based on the absolute value only seemed relatively low and seemed to be difficult to use for the information input system. However, the relative difference in pupil responses under conditions where the spatial frequency of the target icon alternates has not been examined. So, in the following three experiments, subjects were instructed to maintain attention on a target image in a hybrid stimulus whose spatial frequency components alternated periodically between HSF and LSF, and the spatial frequency components of the two icon images in a hybrid stimulus were always different.

The first experiment used hybrid stimuli composed of two static icon images. In the second and third experiments, we employed hybrid stimuli, including dynamic icon image(s), to make two images and the transition of spatial frequency components more salient. The second experiment displayed hybrid images composed of two dynamic icon images, and the third experiment displayed hybrid stimuli composed of static and dynamic icon images. The pupil diameter was continuously recorded using an eye tracker. The pupil changes were analyzed before and after the transition of spatial frequency components to determine which of the two icon images the subject was keeping attention on. Results consistently showed that attending to HSF icon images was associated with smaller pupil sizes, while attending to LSF icon images produced larger pupil responses. Furthermore, pupils constricted when the spatial frequency components of attentional icon images changed from LSF to HSF and dilated when it changed from HSF to LSF. These effects were stronger under the dynamic condition.

To investigate whether attentional targets associated with HSF and LSF components could be inferred from pupil responses, we developed a classification method based on pupil changes before and after the alternation of spatial frequency components of attentional icon image. The proposed method achieved classification accuracy exceeding 70% using healthy subjects, thereby meeting the performance criteria typically required for augmentative and alternative communication (AAC) systems [18]. In addition to the accuracy, the approach base on this technology offers several advantages. The setup is simple, does not require calibration, and assistance from a specialist. It relies on icon images rather than text, which allows for more straightforward communication, and is suitable for users who are using different languages or have limited literacy skills. The glasses-type eye tracker can be applied, allowing use in both sitting and lying positions. It does not require overt motor control, making it promising for individuals with serve physical impairments. The findings in this study show the potential of decoding attention direction based on pupil responses and offering a non-invasive, non-verbal method to support communication.

## Experiment 1: Pupil responses when directing attention to one of the static hybrid images

In Experiment 1, we measured pupil size while the observer was paying attention to one of two icon images in a hybrid stimulus. The hybrid stimulus consisted of two superimposed static icon images filtered to have different spatial frequency components.

### Methods

#### Subjects.

Eight subjects (4 females and 4 males, aged 20–60) with normal or corrected-to-normal vision participated in this experiment. All methods and experimental procedures complied with the Declaration of Helsinki, and experimental protocols were approved by Human Subject Research Ethics Review Committee. All subjects were adults and provided written informed consent on the day of the experiment before participation.

#### Apparatus.

**Fig 1:**
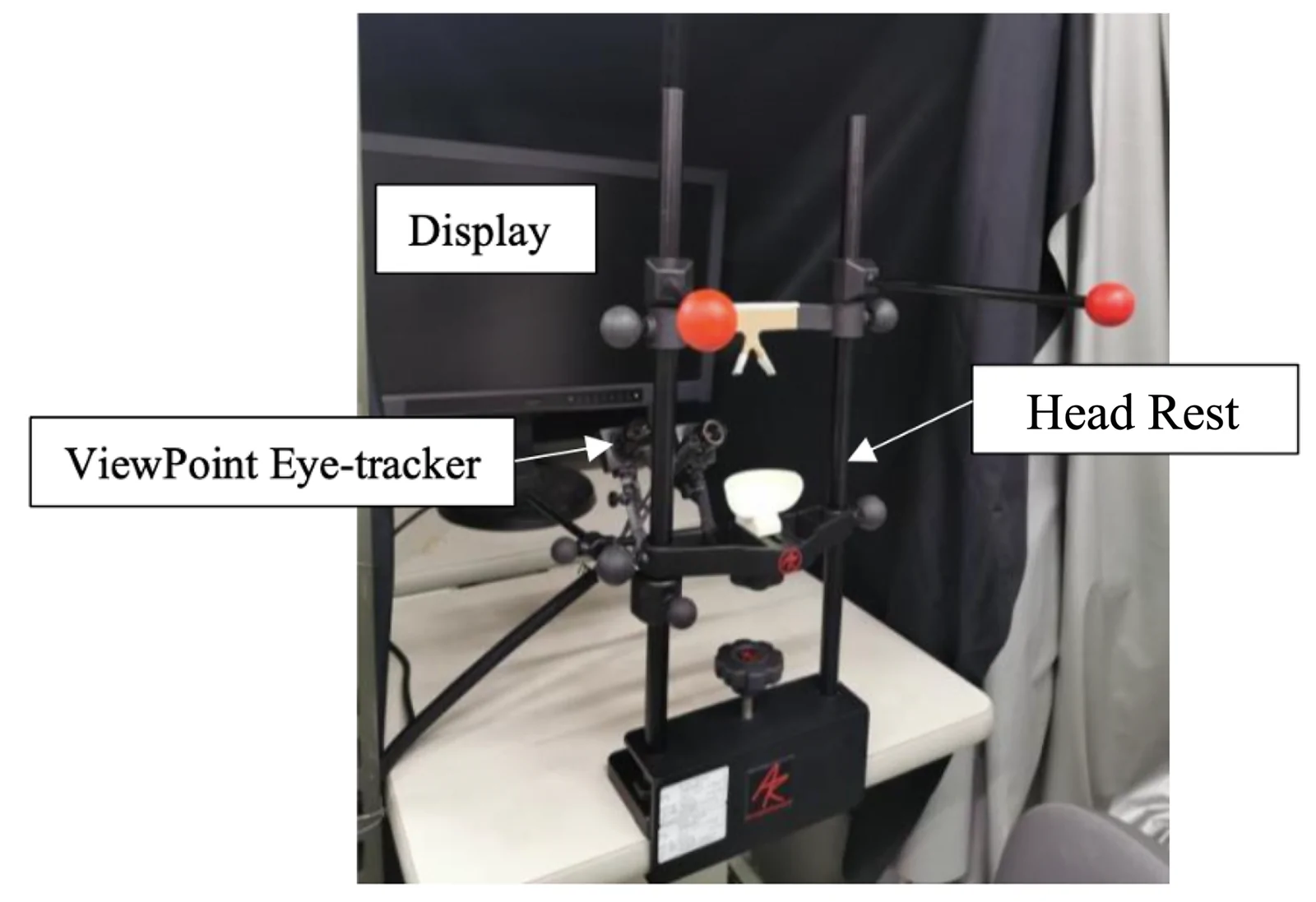
Experiment set-up. The experimental setup used in Experiment 1 that includes an eye-tracker, a head rest, and a display.

Fig 1 shows the experimental setup, which included an infrared eye-tracking system (ViewPoint EyeTracker BHU 903 90 Hz, Arrington Research) that detects pupil diameter changes in a dark environment, which was based on a previous pupillary response-based eye-gaze input system. This is a general method that applied in many related studies, though different kind of Video-oculography system are used [17,19] This system capture eye images using an external camera, detects pupil diameter from eye surface through image processing, and estimates the gaze direction by combining it with calibration data and an eye shape model. The set-up also consisted of an LCD display (ColorEdge CG275W 27.0 inch, EIZO) that presents the stimulus screen at a 90 Hz frame rate, a headrest that kept subjects’ head position stable and a PC (Apple MacBook) that executes a code generated via MATLAB 2018b equipped with Psychophysics Toolbox Version 3 (PTB-3).

#### Visual stimuli.

**Fig 2:**
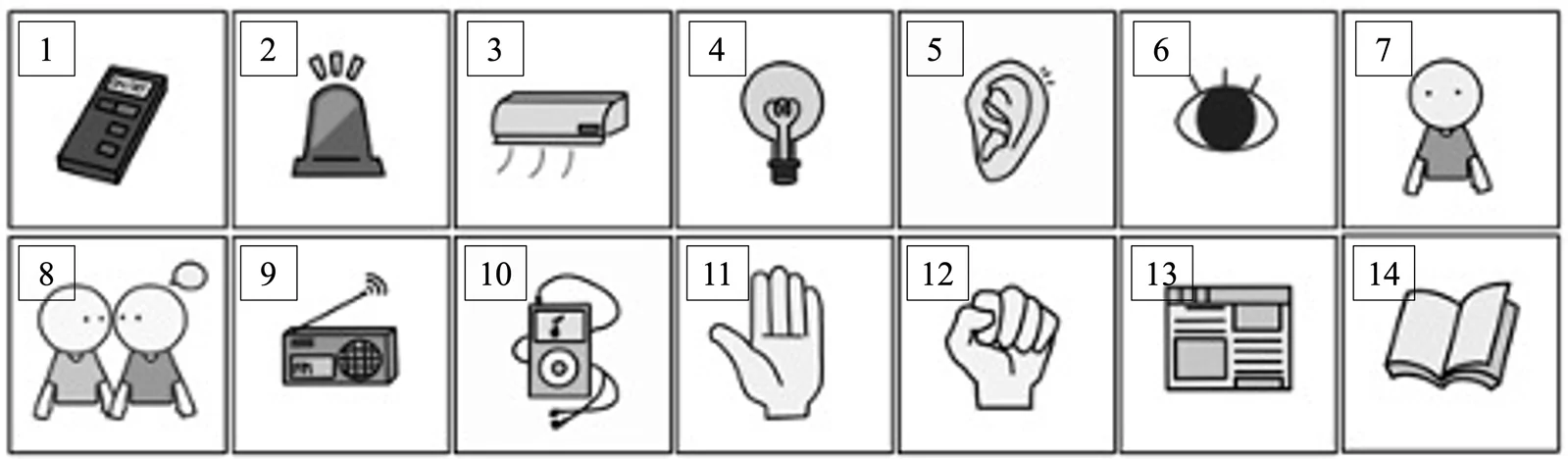
Original icons images. The icons illustrate the possible needs in a communication support system, such as reading a book (icon 14), browsing the webpage (icon 13), turning on/off (icon 11 and 12) the light or the air-conditioner (icon 3 and 4).

**Fig 3:**
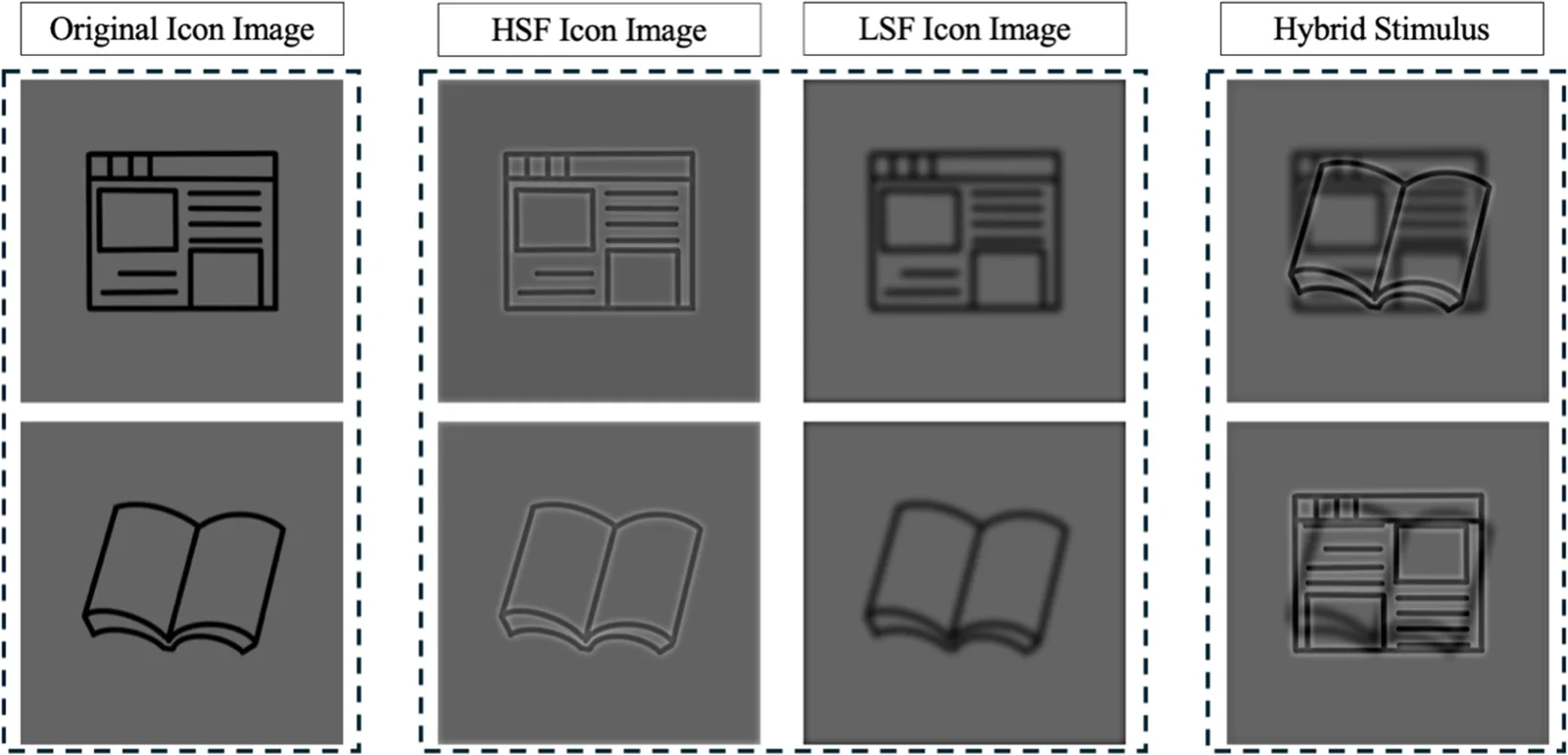
Development of hybrid stimuli. Sample icon images (browsing webpage and reading book) were filtered to contain high- and low-spatial frequency components, which were then combined to generate the corresponding hybrid stimulus used in Experiment 1.

Experiment 1 included 14 hybrid stimuli generated from 14 original icons (7 icon pairs), as shown in Fig 2. The original icons were selected based on assumed user needs, and all icons were designed and illustrated by the author. Fig 3 (left) illustrates the process of creating a hybrid stimulus using the “web browsing” and “reading” icons as an example. A two-dimensional Gaussian filter was applied to each icon image to produce both high-pass and low-pass filtered icon images (Fig 3 (center), following Equation (1) where G(x) represents the value of the Gaussian function at coordinates (x,y), σ is the standard deviation of the Gaussian distribution, and e is the base of the natural logarithm (approximately 2.72) [20]. To improve computational efficiency, the filtering process can be implemented using two one-dimensional convolutions, along the horizontal (x) and vertical (y) axis separately as shown in Equation (2).


$$\begin{array}{c} G(x,y)=\frac{1}{2\pi \sigma^{2}}\exp\;\left(-\frac{x^{2}+y^{2}}{2\sigma^{2}}\right) \end{array}$$ (1)

$$\begin{array}{c} G(x,y)=G(x)\cdot G(y),\;G(x)=\frac{1}{\sqrt{2\pi }\sigma }\exp\;\left(-\frac{x^{2}}{2\sigma^{2}}\right),\;G(y)=\frac{1}{\sqrt{2\pi }\sigma }\exp\;\left(-\frac{y^{2}}{2\sigma^{2}}\right) \end{array}$$ (2)

The Gaussian filter operates by convolving the input image with a Gaussian kernel. Adjusting the standard deviation (σ) controls the degree of blur: larger σ values yield smoother edge transitions and greater image blurriness. The hybrid image stimulus *H* (Fig 3, right) was constructed by combining two images, *I*_1_ and *I*_2_: the first filtered with a low-pass filter $G_{L}$, and the second filtered with a high-pass filter defined as $G_{H}=1-G_{L}$, as described in Equation (3) [21].

Pre-experimental verbal feedback from subjects informed the choice of a standard deviation of 8, as this value enabled clear differentiation between images with HSF and LSF components, while allowing subjects to recognize the content of each image accurately. In the experiment, the combinations of icons in each hybrid stimulus were always the same; for example, icon images of webpage and book were grouped as a hybrid stimulus for selecting the type of entertainment. Each icon pair was then used to create two kinds of hybrid stimuli containing different spatial frequency, HSF or LSF, components for the same icon image, as shown in Fig 3 (the center panel), and then the images with different frequency components were combined to make a hybrid stimulus (the right panel). All hybrid stimuli are grayscale, and their average luminance has been standardized using the SHINE Toolbox [22]. The luminance histogram has been adjusted as well.


$$\begin{array}{c} H=I_{1}*G_{L}+I_{2}*G_{H}=I_{1}*G_{L}+I_{2}*(1-G_{L}) \end{array}$$ (3)

#### Procedures.

**Fig 4:**
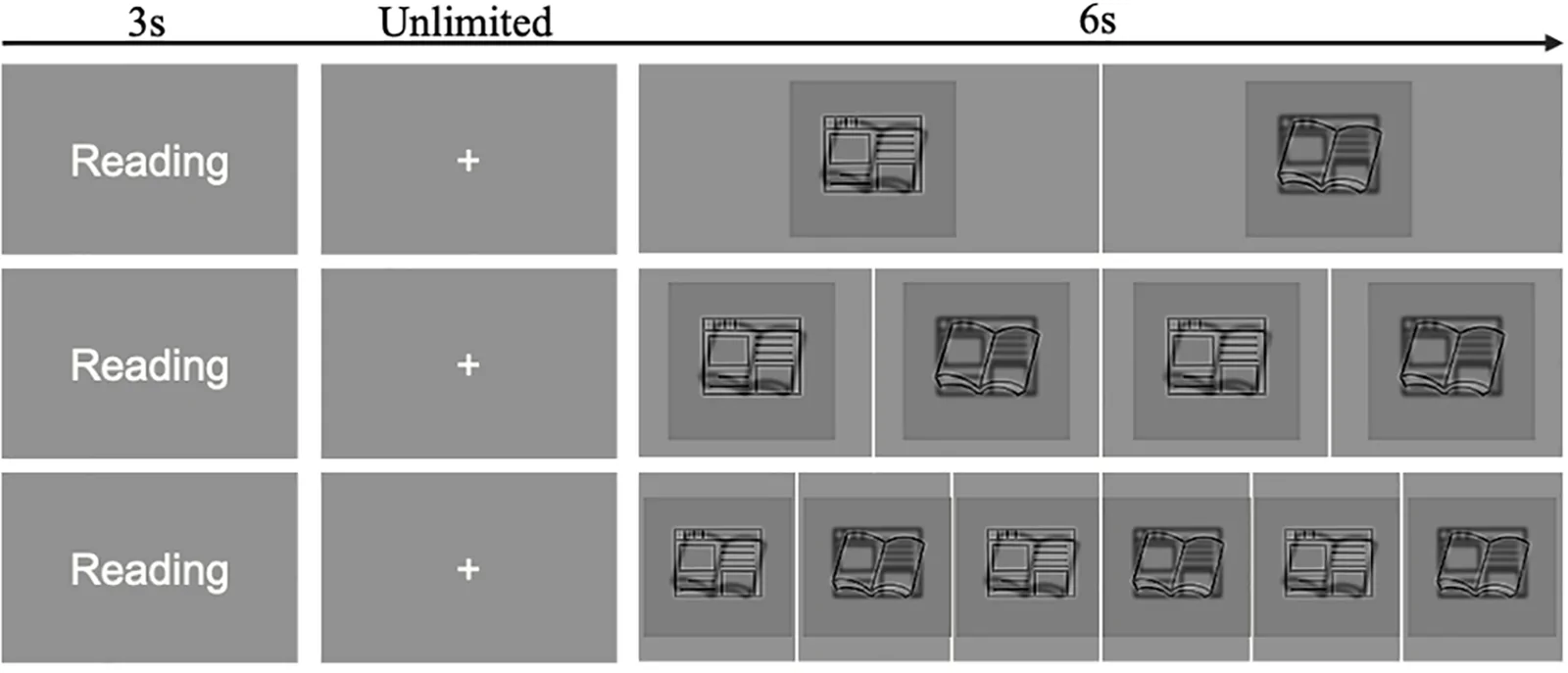
Presentation sequence in Experiment 1. Presentation sequences in one trial for three alternation frequencies in Experiment 1: 0.16 Hz (upper), 0.33 Hz (middle), 0.50 Hz (lower).

Before the experiment, each subject sat in front of the screen and conducted an eye-tracker calibration. The observation distance was 75 cm. Fig 4 outlines the stimulus presentation in each trial. Each trial began with a 3-second instruction screen indicating the icon image to which attention should be directed in the hybrid stimulus and was followed by a fixation point. Then, the subject pressed the button when they were ready, and a hybrid stimulus was presented 500 ms after the button was pressed. The spatial frequency components of two icon images in the hybrid stimulus alternated during the 6-second presentation in opposite phases. Three temporal frequency conditions were used for this alternation: 0.16 Hz, 0.33 Hz, and 0.5 Hz. The subject’s task was to maintain attention on the target icon image indicated on the instruction screen throughout the 6-second stimulus presentation, regardless of the spatial frequency alternation. Pupil data was recorded from the button press until the end of the stimulus presentation. Subjects were instructed to avoid blinking as much as possible during stimulus presentation. They were allowed to take breaks between trials if needed.

The experiment consisted of three sessions. Each session included three alternation frequency conditions (0.16 Hz, 0.33 Hz, 0.50 Hz). The stimulus set consisted of seven pairs of icons, which were combined into seven hybrid images. Under each frequency condition, all hybrid images were presented, and each icon was assigned as the target icon across trials. The alternation order of the spatial frequency components was counterbalanced within each pair. The order of image presentations within each session was randomized, and the entire experiment lasted approximately 60 minutes.

#### Pupil data processing.

Since absolute pupil size varies considerably across individuals, pupil data were normalized relative to a baseline value to enable comparison across trials and subjects, following standard preprocessing recommendations [23]. The baseline value was the average pupil diameter during the 500 ms before stimulus onset.

Samples were labeled as invalid if the recorded diameter was identified as an outlier using an interquartile range (IQR) criterion. Missing samples caused by blinks or transient tracking loss were linearly interpolated when the missing duration was shorter than 300 ms, whereas longer missing segments remained missing. The resulting pupil time series was smoothed using a third-order Savitzky–Golay filter with a window length of up to 21 samples [24]. Trials with data loss exceeding 10% were excluded from the analysis.

In this study, the pupil change relative to the baseline value was computed at each timing following the formula (4). A positive value indicates pupil dilation relative to baseline, whereas a negative value indicates constriction relative to baseline. The ViewPoint EyeTracker pupil unit was arbitrary, and we converted it to mm using an artificial pupil of known diameter at the same setup distance and computed a linear ratio.


$$\begin{array}{c} \text{Pupil Change Relative to Baseline}=P_{t}-P_{\text{baseline}} \end{array}$$ (4)

### Results

**Fig 5:**
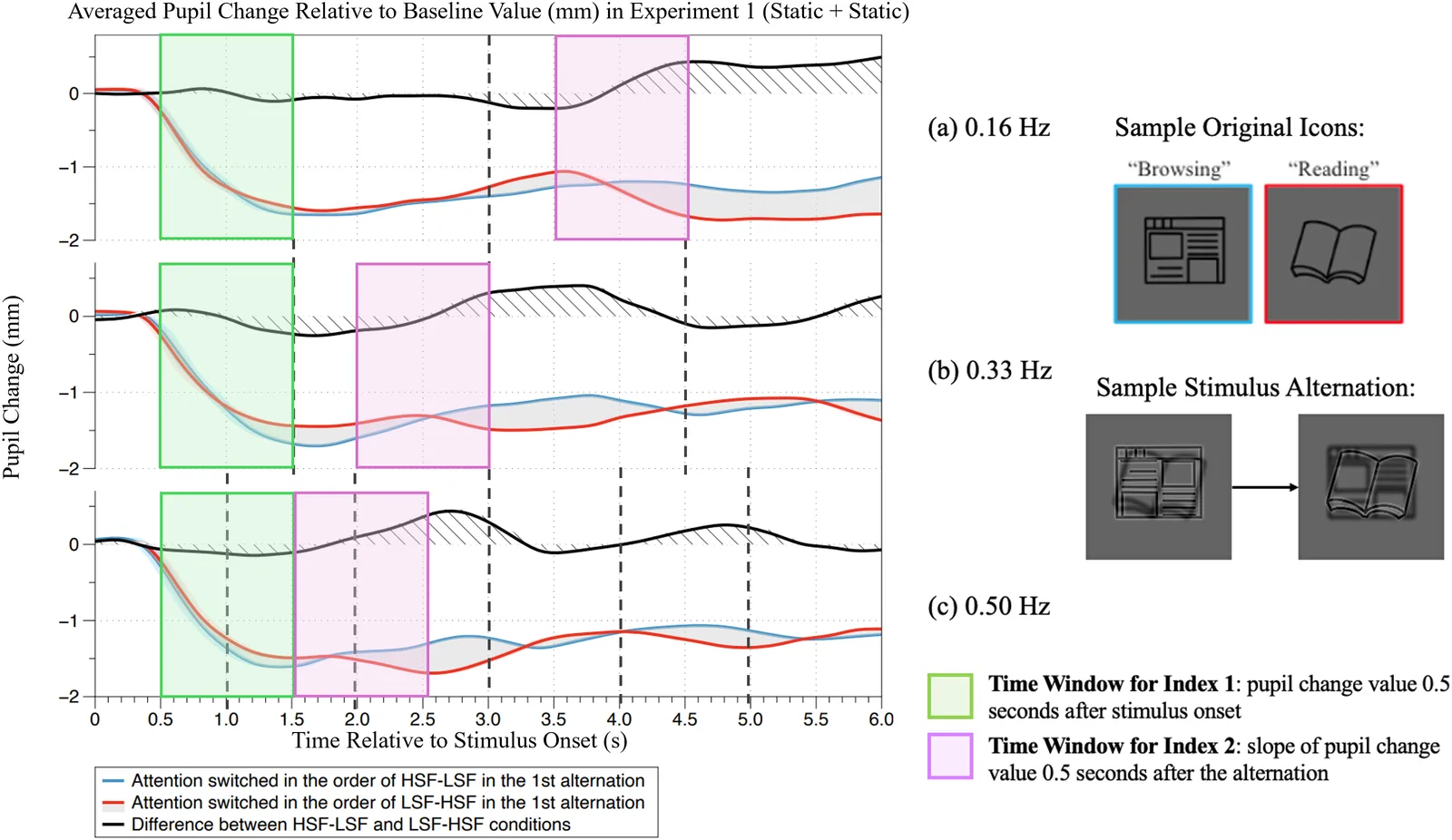
Averaged pupil change in Experiment 1. Average results for the alternation frequency of (a) 0.16 Hz, (b) 0.33 Hz, and (c) 0.50 Hz in Experiment 1. Red lines represent the pupil change relative to the baseline while the initial target image was LSF, and blue lines represent the pupil change while the initial target image was HSF. Black lines represent the differences between those two stimulus conditions. The vertical dashed lines indicate the timing of the image alternation. Sample original icons and the corresponding alterations screen are shown on the right for reference. In this example, the hybrid stimulus consisted of a “reading” icon with low spatial frequency components and a “browsing” icon with high spatial frequency components at the onset of presentation. The green shaded areas represent the time window for the calculation of index (1), and the purple shaded areas represent the time window for the calculation of index (2).

Fig 5 shows the average pupil change value relative to baseline across subjects with the alternation frequency of (a) 0.16 Hz, (b) 0.33 Hz, and (c) 0.50 Hz as a function of time. The red line represents pupil change value when the target icon image was LSF at the beginning and swapped to HSF components after the first alternation, while the blue line represents pupil change value when the target icon was swapped from HSF to LSF components. The black line shows the difference between the blue and red lines. The vertical dash line represents the moment of alternation. Sample icon images on the right are provided for illustration purposes only and the colors of their frames correspond to those of data lines shown in Fig 4. The plotted results reflect the average across all subjects and trials. The data in Fig 5(a) show that, when the hybrid stimulus was presented, the pupil constricted to a minimum value around 1.5 seconds after stimulus onset. Then, in the case of red line, pupil size gradually dilated and after the spatial frequency components of the icon images were alternated at 3.0 seconds from the stimulus onset, the pupil started to constrict about 0.5 seconds from the alternation, kept constricted for about 1 second, and then remained at a constant level thereafter. In the case of the blue line, pupil size gradually dilated after the alternation of spatial frequency components, and no clear constriction is observed. Fig 5(b) and 5(c) show the average results for the hybrid stimulus with the alternation frequencies of 0.33 Hz and 0.50 Hz, respectively. These results indicate that even when the alternation frequency differs, the trends in pupil response after spatial frequency alternation are similar, clearly demonstrating that pupil responses differ depending on the spatial frequency components of the target icon image when observing the same hybrid stimulus.

As shown in Fig 5, pupil size was smaller when subjects focused on the target icon image with the HSF components before the first alternation, which is consistent with the previous report by Hu et al. 2018 [17]. Then, pupil tends to dilate or remain stable when the spatial frequency components of the target icon image are swapped from HSF to LSF (blue line) and constrict when they are swapped from LSF to HSF (red line) after the first alternation. Therefore, attentional targets were identified using two indices: (1) absolute pupil change 0.5–1.5 seconds after onset (defined as the difference between the maximum and minimum pupil size within the window), which is highlighted by the green boxes; and (2) pupil change slope 0.5–1.5 seconds after alternation (defined as the slope of a linear regression fitted to the pupil time series with the window). Purple boxes highlight the time windows following the vertical dashed line, indicating the timing of pupil reactions to stimulus alternation in Fig 5.

For the first index, the absolute pupil change before alternation (0.5–1.5 s), paired-samples *t*-tests comparing the HSF and LSF attentional target conditions revealed no statistically significant differences at any alternation frequency. Specifically, no significant differences were observed at 0.16 Hz (*t*(7)  =  0.26, *p*  =  0.802), 0.33 Hz (*t*(7)  =  2.18, *p*  =  0.066), or 0.50 Hz (*t*(7)  =  0.81, *p*  =  0.445). Cohen’s $d_{z}$ indicated a negligible effect size at 0.16 Hz ($d_{z}=0.09$), a medium-to-large effect size at 0.33 Hz ($d_{z}=0.77$), and a small effect size at 0.50 Hz ($d_{z}=0.29$). Although the 0.33 Hz condition exhibited the largest effect size among the three frequencies, the difference did not reach statistical significance. These results suggest that pupil change before alternation alone is a relatively weak indicator for identifying the attentional target in hybrid stimuli.

The second index was the pupil change slope measured over a 1-second time window beginning 0.5 seconds after the first alternation. Because the stimulus duration was fixed at 6 seconds, only the 0.33 Hz and 0.50 Hz conditions contained multiple alternations within a single trial, allowing pupil responses after the second and third alternations to be examined. A 3 (time window: after the first, second, or third alternation) × 2 (alternation direction: HSF to LSF or LSF to HSF) repeated-measures ANOVA was performed separately for the 0.33 Hz and 0.50 Hz conditions. No significant interaction between time window and alternation direction was found at either 0.33 Hz (*F*(2,14)  =  1.88, *p*  =  .189) or 0.50 Hz (*F*(2,14)  =  2.35, *p*  =  .132), indicating that the effect of alternation direction on pupil change slope did not significantly differ across successive alternations. Therefore, subsequent analyses focused on the pupil change slope after the first alternation.

**Fig 6:**
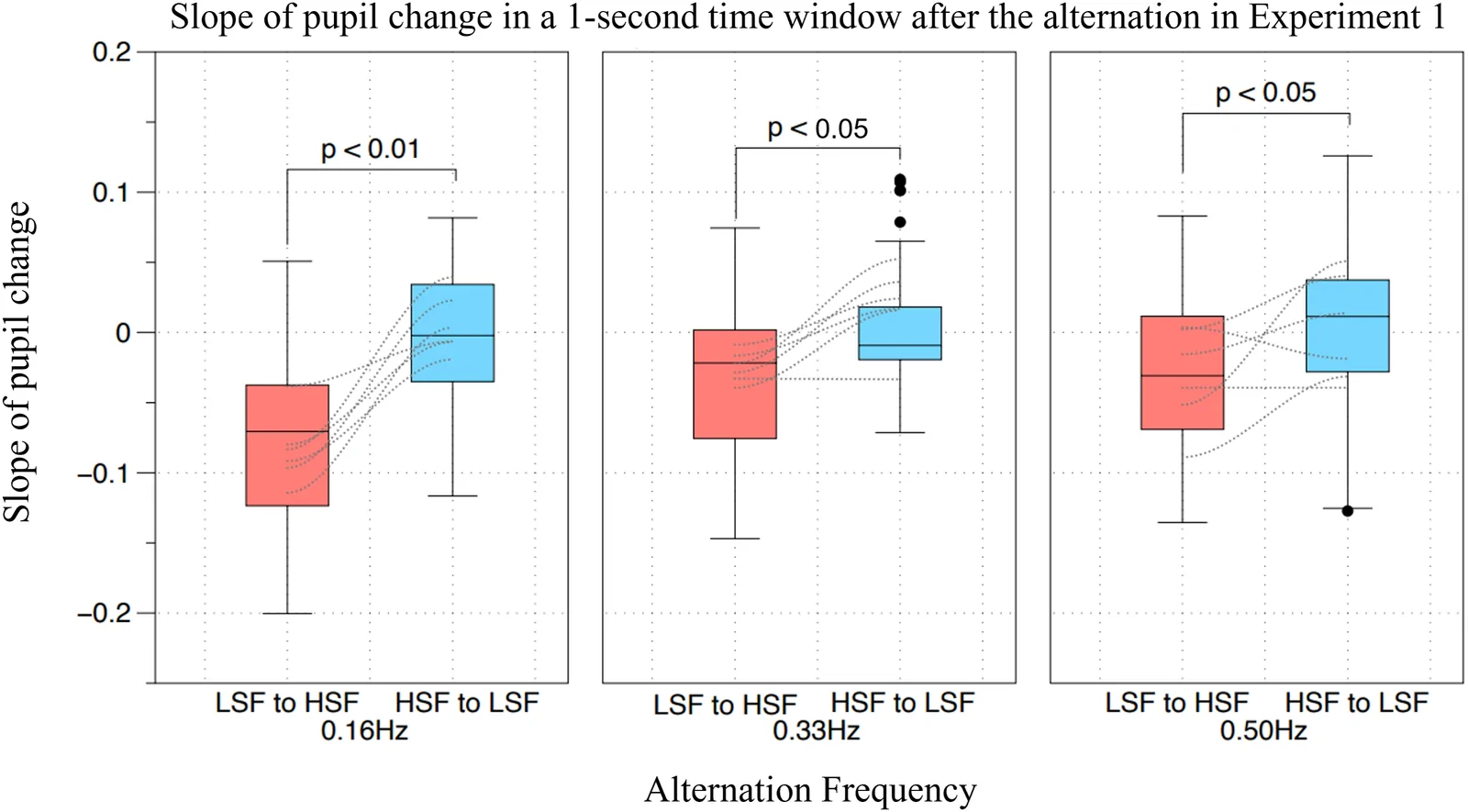
The box plot shows the pupil changed slope after the first alternation in Experiment 1. Pupils change slope in a 1-second time window after alternation for alternation frequencies of 0.16 Hz (left), 0.33 Hz (middle), and 0.50 Hz (right). Red boxes indicate the slopes of pupil change when the spatial frequency components of the target icon images were alternated from LSF to HSF, and blue boxes indicate those when they were alternated from HSF to LSF. In each boxplot, the central line indicates the median, and the box represents the interquartile range (25th–75th percentiles). Whiskers extend to 1.5 times the interquartile range, and dots indicate outliers. The gray dashed lines connect the results obtained from the same subject. Statistical analyses were performed using paired-samples t-tests.

The three panels in Fig 6 illustrate the distribution of pupil change slopes across all subjects within a 1-second time window starting 0.5 seconds after the first stimulus alternation, for the 0.16 Hz (left), 0.33 Hz (middle), and 0.50 Hz (right) conditions, respectively. In each panel, red boxplots represent the pupil change slopes when the target alternated from LSF to HSF, whereas blue boxplots represent the slopes for alternations from HSF to LSF. Across all frequency conditions, the distributions of slopes for the two alternation directions are clearly separated, with consistent differences between the red and blue boxplots, suggesting a systematic effect of alternation direction on pupil dynamics.

To evaluate the significance of these differences, paired-samples *t*-tests were conducted between the HSF-to-LSF and LSF-to-HSF conditions for each alternation frequency. Significant differences were observed at all frequencies: 0.16 Hz (*t*(7)  =  4.13, *p*  =  0.004), 0.33 Hz (*t*(7)  =  3.01, *p*  =  0.020), and 0.50 Hz (*t*(7)  =  2.42, *p*  =  0.046). In addition, all conditions demonstrated large effect sizes ($d_{z}=1.46$, 1.07, and 0.86 for 0.16 Hz, 0.33 Hz, and 0.50 Hz, respectively), suggesting that alternation of spatial frequency components significantly enhanced the differences in pupil responses when attending to HSF versus LSF target images.

To further examine whether the pupil slopes reflected systematic pupil changes, one-sample *t*-tests against zero were additionally conducted for each condition. In the LSF-to-HSF condition, the slope values were significantly smaller than zero at 0.16 Hz (t(7)=−7.08, *p* < 0.001) and 0.33 Hz (t(7)=−2.76, *p*  =  0.028), indicating a reliable pupil constriction trend following the alternation from LSF to HSF components. In contrast, no significant deviations from zero were observed in the HSF-to-LSF condition or under the 0.50 Hz condition.

These results indicate that pupil dynamics induced by alternation of spatial frequency components can be utilized to infer the attended icon across all tested frequencies, with the strongest discriminative effect observed at 0.16 Hz.

## Experiment 2: Pupil responses when directing attention to a dynamic hybrid stimulus

This experiment aimed to examine whether adding motion to icon images could increase the difference in pupil responses when observing the target icons with different frequency components in a hybrid stimulus. We assume that, in this dynamic stimulus condition, the difference in pupil responses to different spatial frequency components increases because motion helps to maintain their visibility and draws attention strongly.

### Methods

#### Subjects.

Seven subjects (3 females and 4 males, aged 20–60) with normal or corrected-to-normal vision participated; 5 of them had also participated in Experiment 1. Subjects were recruited in the laboratory between February 2024 and March 2024. All methods and experimental procedures complied with the Declaration of Helsinki, and experimental protocols were approved by Human Subject Research Ethics Review Committee. All subjects were adults and provided written informed consent on the day of the experiment before participation.

#### Apparatus.

An infrared eye-tracking system (EyeLink 1000, SR Research) was used. The system provided a spatial resolution of 0.1% of a 5 mm artificial pupil and sampled data at 250 Hz. Visual stimuli were presented on a CRT display (Sony GDM-F500R, 1280 × 1024 pixels, 60 Hz refresh rate). The experimental code was developed using MATLAB 2018b equipped with the Psychophysics Toolbox Version 3 (PTB-3).

#### Visual stimuli.

**Fig 7:**
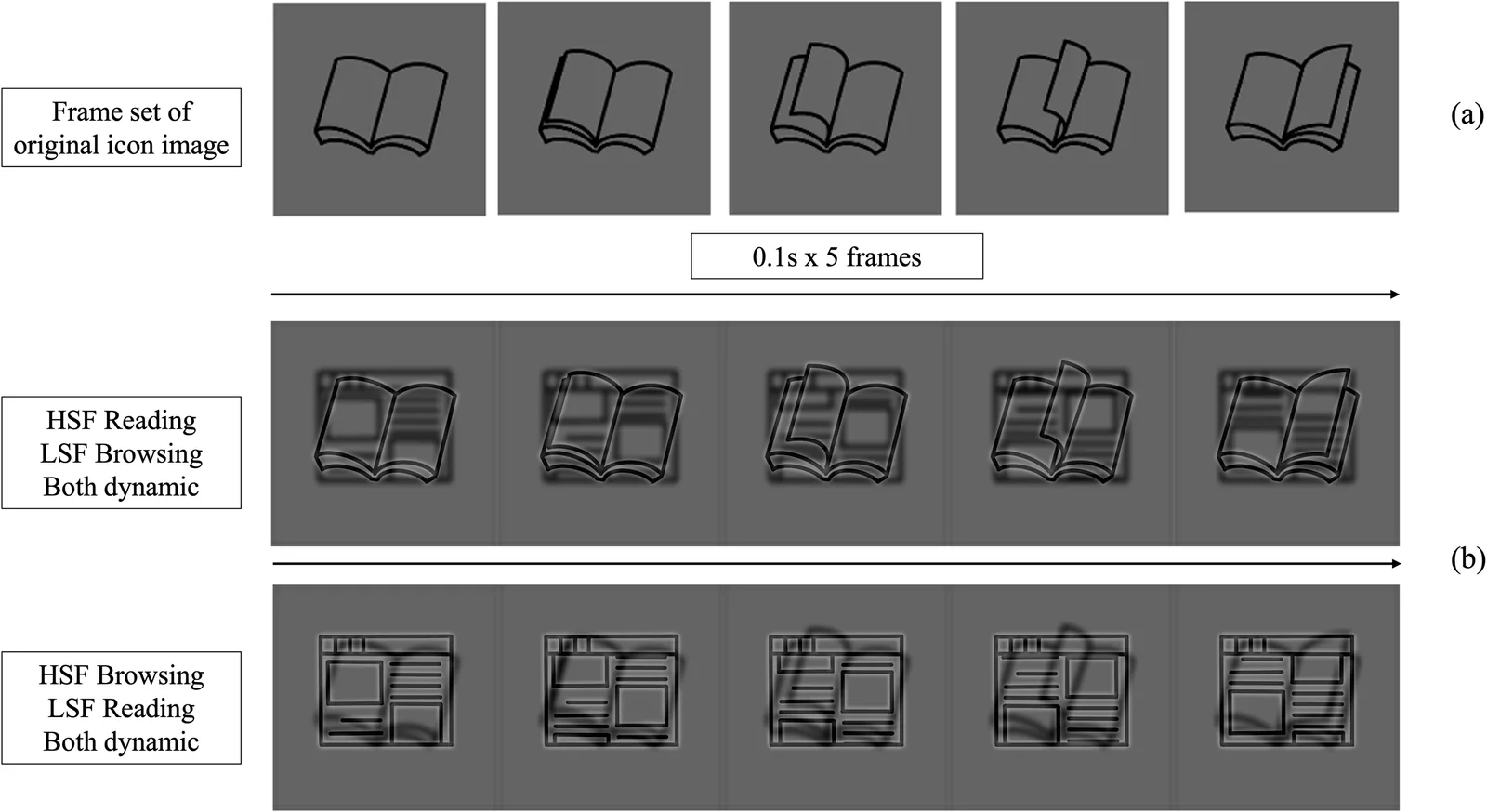
Sample frame set of dynamic icon and hybrid image used in Experiment 2. (a) Each dynamic icon image consisted of five frames, with each frame displayed for 0.1 seconds and looped. (b) Two icon images were filtered and combined to generate a hybrid image. Each dynamic hybrid image consisted of five hybrid frames, with each frame displayed for 0.1 seconds and looped. Both HSF and LSF components were dynamic.

Similar to Experiment 1, hybrid stimulus composed of two icon images with HSF and LSF components was used as the stimulus. Here, 14 original icons were filtered, added motion information, and grouped as 7 pairs, and each pair was combined to generate two dynamic hybrid stimuli with opposite spatial frequency conditions. Since some of the icon images used in Experiment 1 were difficult to represent in dynamic form, such icon images were modified or replaced to ensure clarity and perceptual consistency. Besides, one pair of stimuli used in Experiment 1 was excluded because most subjects reported difficulty selectively attending to the target icon while ignoring the distractor icon under the dynamic presentation condition. As shown in Fig 7(a) each dynamic icon image consists of 5 frames, with each frame displayed for 0.1 seconds to produce an animation effect through continuous presentation. All frames are grayscale, and their average luminance has been standardized using the SHINE Toolbox [22]. The luminance histogram has been adjusted. Fig 7(b) depicts a sample hybrid stimulus consisting of two dynamic icon images.

#### Procedures.

**Fig 8:**
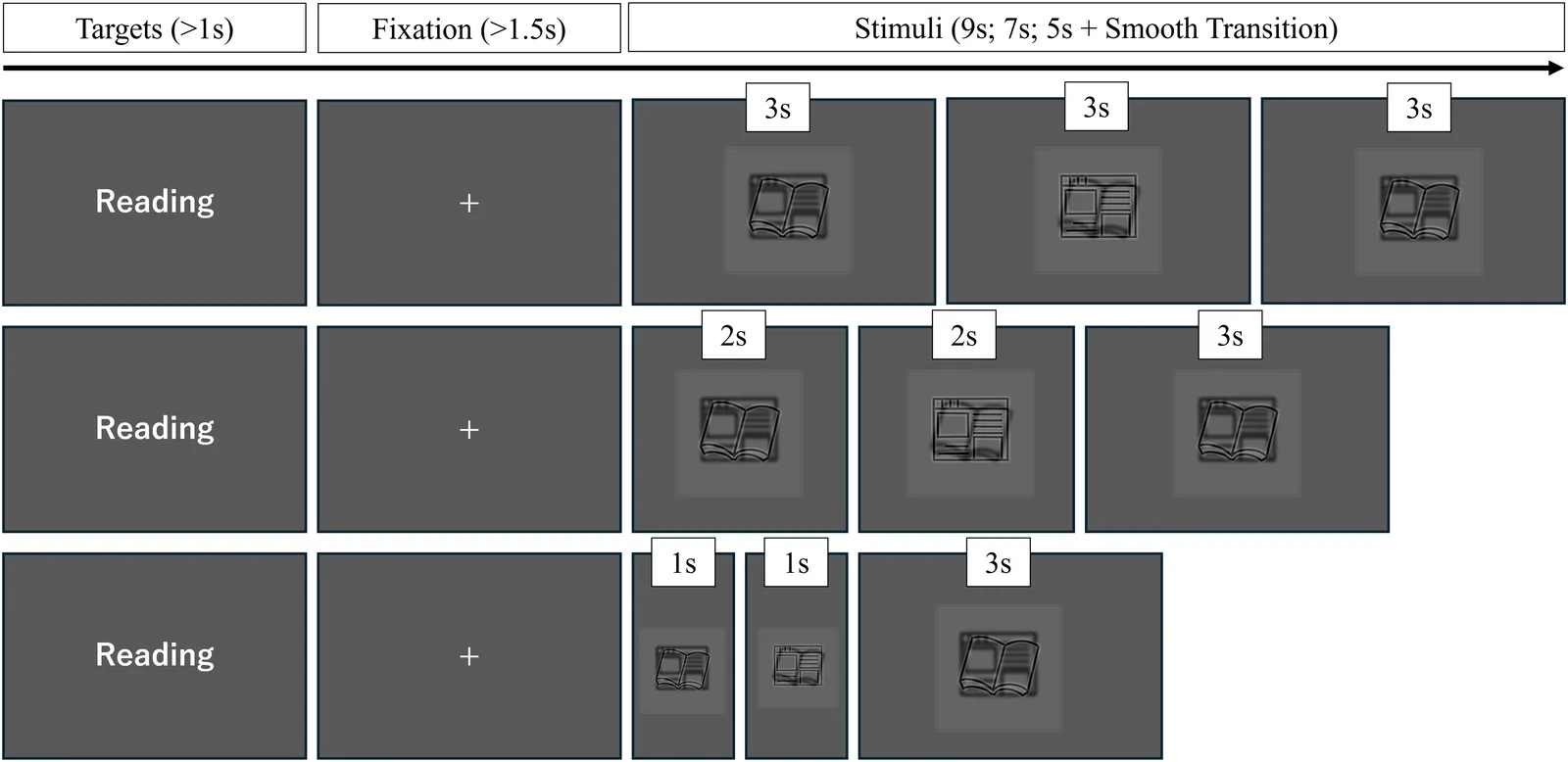
Presentation sequence in Experiment 2. Presentation sequences in one trial for three alternation frequencies in Experiment 2: 0.16 Hz (upper), 0.25 Hz (middle), 0.50 Hz (lower). See the main text for the details.

The task of Experiment 2 was identical to that in Experiment 1, but the trial structure differed. The trial duration in Experiment 1 was fixed as 6 seconds to maintain overall exposure time across conditions but led to more alternations in the higher alternation frequency condition. However, no significant interaction was found between time window and alternation direction within either the 0.33 Hz or 0.50 Hz condition. This suggests that extending trial duration in the low-frequency condition did not provide additional information but may have introduced unnecessary variability due to fatigue or attentional lapses. The following experiments instead fixed the number of alternations to two per trial for a better experience. As shown in Fig 8, each trial began with an instruction screen indicating the target icon image, followed by a fixation point. After subjects pressed the button when they were ready (available at least 1.5 seconds later from the end of the previous trial), the hybrid stimulus was presented 500 ms after the button was pressed. Subjects were instructed to focus on the target image throughout the trial. Three conditions were used for the alternation of spatial frequency components of the icon images: 0.16 Hz, 0.25 Hz, and 0.50 Hz, with each trial including two alternations of spatial frequency components in the icon image. The last alternated stimulus was always displayed for 3 seconds to ensure the pupil response could be fully recorded. A 500 ms fade-in/out phase was added at each alternation to ensure a smooth transition of images and shift of attention. The display time for each single trial was 10, 8, and 6 seconds for the alternation frequencies 0.16 Hz, 0.25 Hz, and 0.50 Hz. The experiment consists of 84 trials for each alternation frequency condition, resulting in 252 trials for each subject.

#### Pupil data processing.

The pupil data processing in Experiment 2 was identical to that in Experiment 1.

### Results

**Fig 9:**
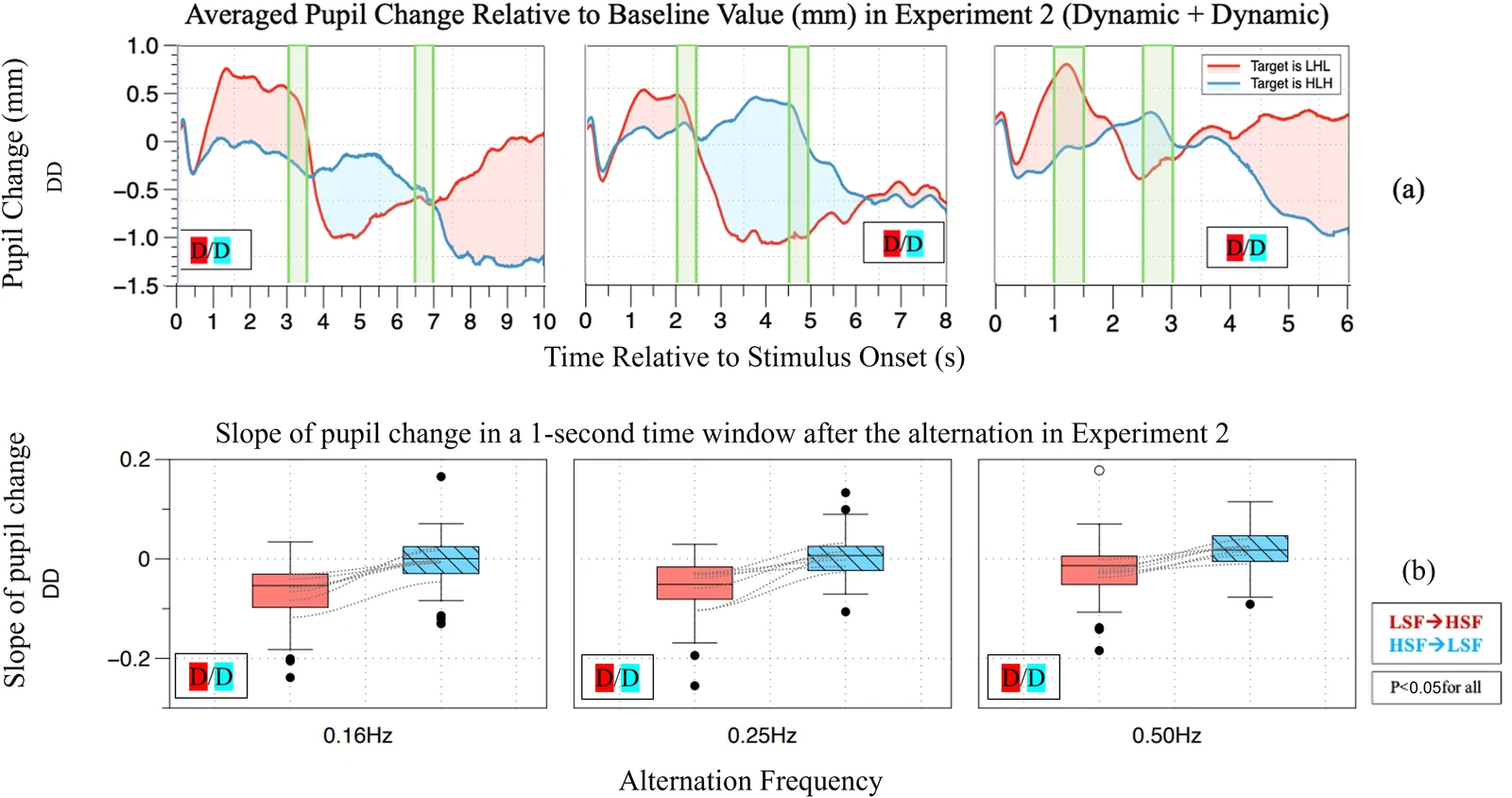
Averaged pupil change and the pupil change slope after the first alternation in box plot in Experiment 2, which is formed by 2 dynamic icons. (a) Average results for the alternation frequency of 0.16 Hz (left), 0.25 Hz (middle), and 0.50 Hz (right) in Experiment 2. Red lines represent the pupil change value while the initial target image was LSF components, and blue lines represent the pupil change value while the initial target image was the HSF components one. The vertical shaded areas indicate the fade-in/out phase of the image alternation. D/D in each panel represents the motion condition of two overlapped icon images, which means the hybrid stimuli in this experiment was formed by two Dynamic icon images. (b) Pupil change slope in a 1-second time window after the first alternation in Experiment 2. Red boxes indicate the slopes of pupil change while the spatial frequency components of target icon images were alternated from LSF to HSF, and blue boxes indicate those while the spatial frequency components of the target icon images were alternated from HSF to LSF. In each boxplot, the central line indicates the median, and the box represents the interquartile range (25th–75th percentiles). Whiskers extend to 1.5 times the interquartile range, and dots indicate outliers. The gray dashed lines connect the results obtained from the same subject. Paired-samples t-tests were conducted to test for statistical significance.

The average pupil change value relative to the baseline value across all subjects was presented in Fig 9(a) for the alternation frequencies of 0.16 Hz (left), 0.25 Hz (middle), and 0.50 Hz (right) as a function of time from the stimulus onset. The average pupil size during 500 ms before the stimulus onset was calculated as the baseline value. The red line shows the pupil response when the initial spatial frequency components of the target image were LSF components and alternated in a sequence of LSF-HSF-LSF, while the blue line shows the pupil response to that of the target image alternated with HSF-LSF-HSF sequence. The timings of stimulus alternation were highlighted as the green shadow areas in the plot, representing the fade-in/out phase.

When subjects attended to the LSF components (red line) after stimulus onset, greater pupil dilation was observed. Following the initial response, distinct pupillary changes were observed after the stimulus alternation. Specifically, pupil dilation occurred when the spatial frequency components of the target image changed from HSF to LSF (blue line), while constriction followed the alternation from LSF to HSF (red line). This trend was consistent across all alternation frequencies (0.16, 0.25, and 0.50 Hz).

The data analysis methods were the same as in Experiment 1 and included two indices: (1) absolute pupil change 0.5–1.5 seconds after onset and (2) pupil change slope 0.5–1.5 seconds after alternation. For the first index, paired-samples t-tests were conducted to compare the average pupil change when subjects directed their attention to either HSF or LSF target images under different frequency conditions. No significant differences were observed under any alternation frequency. At 0.16 Hz, the difference approached statistical significance (*t*(6)  =  2.27, *p*  =  .064). Similarly, no significant differences were observed at 0.25 Hz (*t*(6)  =  1.73, *p*  =  .134) or 0.50 Hz (*t*(6)  =  2.38, *p*  =  .055), although the effect at 0.50 Hz also approached significance.

Paired-samples *t*-tests were also conducted to compare the pupil change slopes after the first alternation phase (the second index) between the two alternation directions (HSF-to-LSF vs. LSF-to-HSF) at each alternation frequency. Significant differences were observed under all frequency conditions. For the 0.16 Hz condition, pupil change slopes differed significantly between the two alternation directions (*t*(6)  =  5.68, *p*  =  .001, $d_{z}=2.15$). Similar significant differences were observed at 0.25 Hz (*t*(6)  =  4.75, *p*  =  .003, $d_{z}=1.80$) and 0.50 Hz (*t*(6)  =  6.68, *p* < .001, $d_{z}=2.53$). The large effect sizes indicate *t*hat the alternation direc*t*ion of target spatial frequency strongly influenced pupil dynamics across all alternation frequencies.

Fig 9(b) presents a box plot showing the pupil change slope in 1 second following the first alternation relative to baseline for the alternation frequency conditions of 0.16 Hz, 0.25 Hz, and 0.50 Hz. Red boxes indicate pupil changes while the spatial frequency condition of the attentional target icon was alternated from LSF to HSF components, and blue boxes represent the condition of alternation of target spatial frequency from HSF to LSF.

Since all alternation conditions in Experiment 2 included two alternations, the pupil slope after the second alternation was also analyzed. Paired-samples *t*-tests were conducted for each alternation frequency condition using the subject-level mean pupil slopes. Significant differences were observed for all three alternation frequencies, including 0.16 Hz (*t*(6)  =  2.65, *p*  =  .038, $d_{z}=1.00$), 0.25 Hz (*t*(6)  =  3.54, *p*  =  .012, $d_{z}=1.34$), and 0.50 Hz (*t*(6)  =  2.92, *p*  =  .027, $d_{z}=1.10$).

These findings indicate that spatial frequency alternation continued to significantly influence pupil responses after the second alternation. However, both the statistical significance and effect sizes were smaller than those observed after the first alternation, suggesting that pupil responses immediately following the first alternation provide a more reliable basis for attention inference. Therefore, only the data following the first alternation were used in the subsequent analyses.

To further examine whether the pupil slopes reflected systematic pupil changes, two-tailed one-sample *t*-tests against zero were conducted using the subject-level mean pupil slopes for each alternation condition. In the LSF-to-HSF condition, the pupil slopes were significantly lower than zero at all alternation frequencies: 0.16 Hz (t(6)=−5.96, *p*  =  .001, $d_{z}=-2.25$), 0.25 Hz (t(6)=−4.41, *p*  =  .005, $d_{z}=-1.67$), and 0.50 Hz (t(6)=−5.10, *p*  =  .002, $d_{z}=-1.93$). These results indicate a consistent pupil constriction trend following the alternation from LSF to HSF components. In the HSF-to-LSF condition, the pupil slopes did not differ significantly from zero at 0.16 Hz (t(6)=−0.51, *p*  =  .627, $d_{z}=-0.19$) or 0.25 Hz (*t*(6)  =  0.32, *p*  =  .758, $d_{z}=0.12$). However, the pupil slopes were significantly greater than zero at 0.50 Hz (*t*(6)  =  3.00, *p*  =  .024, $d_{z}=1.13$), indicating a systematic pupil dilation trend under this frequency condition.

These findings demonstrate that the direction of spatial frequency alternation produced distinct patterns of pupil dynamics. The LSF-to-HSF alternation consistently elicited pupil constriction across all frequencies, whereas the HSF-to-LSF alternation produced a significant dilation trend only at 0.50 Hz. These direction-dependent responses support the potential use of pupil dynamics for classifying the attentional target.

## Experiment 3: Pupil responses when directing attention to a hybrid stimulus composed of dynamic and static images

This experiment aimed to investigate whether the combination of static and dynamic images could increase the differences in pupil responses when observing the target icons with different components in a hybrid stimulus. We assume that the presence and absence of motion in the icon images may enhance the difference in pupil responses because of the motion contrast of icon images.

### Methods

The subjects and apparatus in Experiment 3 were identical to those in Experiment 2.

#### Visual stimuli.

**Fig 10:**
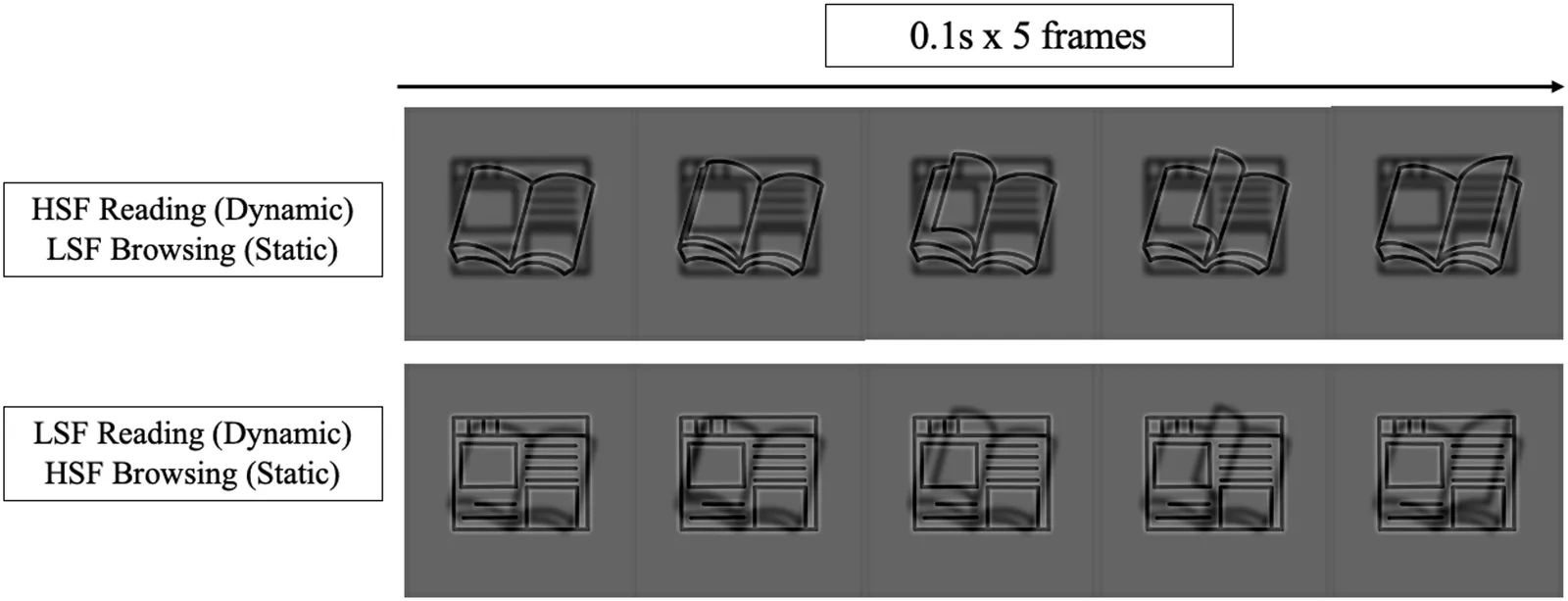
Sample frame set of a dynamic/static hybrid stimulus used in Experiment 3. Each dynamic/static hybrid image consisted of five frames, with each frame displayed for 0.1 seconds and looped. One icon image was dynamic, and the other static. The upper panel shows a sample frame set with a dynamic HSF and a static LSF icon, while the lower panel shows the reverse combination with a dynamic LSF and a static HSF icon.

Similar to Experiments 1 and 2, a hybrid stimulus composed of two icon images with HSF and LSF components was used. Each icon image was filtered respectively to retain either high or low spatial frequency components, and the two icon images with different spatial frequency components were presented and alternated in inverse phase as in Experiments 1 and 2. In Experiment 3, each hybrid stimulus was formed by combining one dynamic target icon image with one static target icon image. The attentional target in each trial was always the same icon image in either a dynamic or static state. One condition of hybrid stimulus was a combination of a dynamic image containing HSF components and a static image containing LSF components at the start of the trial, and the other condition was the reverse combination. Fig 10 illustrates an example of the frames of a hybrid image consisting of a dynamic ’reading’ icon and a static ’browsing’ icon. For each pair of icon images, there were two combinations of icon images for this presentation method, and the attentional target was either a dynamic or static icon image for each hybrid stimulus. Each dynamic icon consisted of 5 frames, each presented for 0.1 seconds to create a continuous motion effect, as in the dynamic icon image in Experiment 2. All frames were presented in grayscale, and their average luminance was standardized using the SHINE Toolbox, and the luminance histograms were equalized to minimize low-level visual confounds.

#### Procedure.

The procedures of Experiment 3 were the same as Experiment 2, which began with an instruction screen indicating the target icon image, followed by a fixation point. The hybrid stimulus consisted of two icon images with opposite spatial frequency components, which alternated in different frequencies: 0.16 Hz, 0.25 Hz, and 0.50 Hz, while the motion condition of each icon image, dynamic or static, remained fixed throughout the trial. Subjects were instructed to always focus only on the same image. Each pair of icon images yielded four presentation combinations, and either icon could serve as the target. This resulted in 56 unique trials (7 pairs × 4 combinations × 2 target icons). Each participant completed 504 trials in total (56 trials × 3 alternation frequencies × 3 sessions).

#### Pupil data processing.

The pupil data processing in Experiment 3 was identical to that in Experiments 1 and 2.

### Results

**Fig 11:**
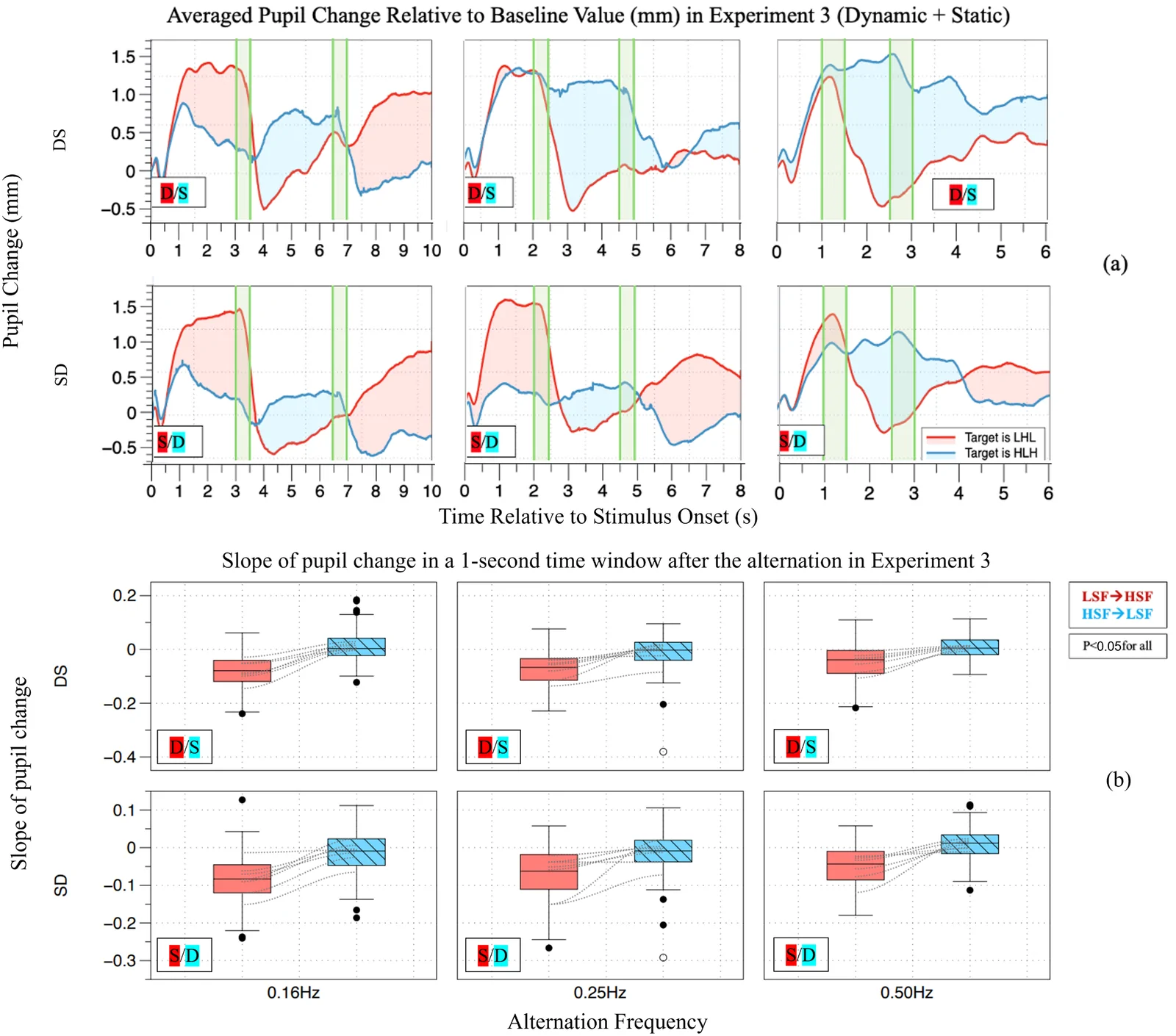
Averaged pupil change and the pupil change slope after the first alternation in box plot in Experiment 3. (a) Average pupil change relative to baseline value for the alternation frequency of 0.16 Hz (left), 0.25 Hz (middle), and 0.50 Hz (right) in Experiment 3. Red lines represent the pupil change value while the initial target image was LSF components, and blue lines represent the pupil change value while the initial target image was the HSF components. The motion condition for the target icon in red lines was always dynamic in the top panel and static in the bottom panel. On the other hand, in the blue lines, the motion condition was always static in the top panel and dynamic in the bottom panel. (b) The slope of pupil change after the first alternation. Red boxes indicate the slope of pupil change while the spatial frequency components of target icon images were alternated from LSF to HSF, and blue boxes indicate the slope of pupil change while those of target icon images were alternated from HSF to LSF. The label D/S represents the combination of the hybrid image, which is formed by a Dynamic icon image and a Static icon image. The motion condition for the target icon in red boxes was always dynamic in the top panel and static in the bottom panel. However, in blue boxes, the motion condition was always static in the top panel and dynamic in the bottom panel. In each boxplot, the central line indicates the median, and the box represents the interquartile range (25th–75th percentiles). Whiskers extend to 1.5 times the interquartile range, and dots indicate outliers. The grey dashed lines connect the results obtained from the same subject. Statistical analyses were performed using paired-samples t-tests.

Fig 11(a) shows the average pupil change relative to the baseline value across all subjects as a function of time from the stimulus onset. The upper panels show the results for the hybrid stimulus containing a dynamic icon image starting with LSF components, which refers to a stimulus composed of dynamic (SF sequence: L-H-L) and static (SF sequence: H-L-H) icon images. The lower panels show those for the hybrid stimulus containing a dynamic icon image starting with HSF components, which refers to a stimulus composed of dynamic (SF sequence: H-L-H) and static (SF sequence: L-H-L) icon images. In each hybrid stimulus, the target icon was either a dynamic or static icon image. The results for the alternation frequency conditions of 0.16 Hz, 0.25 Hz, and 0.50 Hz are plotted in the left, center, and right panels in each row, respectively. In all conditions, the red line represents the results of trials where the spatial frequency components of the attentional target started with LSF followed by HSF and LSF alternation sequence, while the blue line represents those that started with HSF followed by LSF and HSF alternation sequence.

Across all combinations, pupil dilation was observed right after the onset of the hybrid stimulus. The pupil size changes after the onset and after the alternation of spatial frequency components were considered. These trends were consistent with those of Experiments 1 and 2. As shown in Fig 11(a), after the onset of stimulus, pupils dilated more when subjects focused their attention on the LSF image in many cases (red line). After the first alternation of spatial frequency components, pupil constriction occurred when the attentional target changed from LSF to HSF (red line), whereas pupil size was either stable or slightly increased when the attentional target changed from HSF to LSF (blue line). Compared to Experiment 2, the amplitude of pupil dilation in Experiment 3 seemed greater overall.

The pupil change value relative to baseline was calculated for the before-alternation interval. In the condition containing a dynamic icon image starting with LSF components, significant differences between the HSF and LSF target conditions were observed at 0.16 Hz and 0.50 Hz. Specifically, a significant difference was found at 0.16 Hz (*t*(6)  =  3.26, *p*  =  .017), whereas no significant difference was observed at 0.25 Hz (*t*(6)  =  0.24, *p*  =  .818). A significant difference was again observed at 0.50 Hz (*t*(6)  =  2.69, *p*  =  .036), suggesting that the LSF target tended to elicit a larger pupil dilation than the HSF target before the first alternation.

In the condition containing a dynamic icon image starting with HSF components, a significant difference between the HSF and LSF target conditions was observed at 0.25 Hz (*t*(6)  =  3.78, *p*  =  .009). Similarly, a significant difference was also observed at 0.50 Hz (*t*(6)  =  2.95, *p*  =  .026). In contrast, no significant difference was found at 0.16 Hz (*t*(6)  =  1.80, *p*  =  .122).

The slopes of pupil change were then calculated for the after-alternation phase. For the condition with dynamic icon images starting with LSF components (upper panels in Fig 11(b)), paired-samples *t*-tests revealed significant differences in pupil change slopes between the LSF to HSF and HSF to LSF alternation sequences across all alternation frequencies. At 0.16 Hz, a significant difference was observed (*t*(6)  =  7.30, *p* < .001, $d_{z}=2.76$). Similar results were obtained at 0.25 Hz (*t*(6)  =  4.15, *p*  =  .006, $d_{z}=1.57$) and 0.50 Hz (*t*(6)  =  4.97, *p*  =  .003, $d_{z}=1.88$) after the first al*t*ernation.

Moreover, the pupil change slopes after the second alternation were also analyzed. At 0.16 Hz, the LSF-to-HSF condition induced significantly greater pupil constriction than the HSF-to-LSF condition (*t*(6)  =  2.78, *p*  =  .032, $d_{z}=1.05$). Although the same trend was observed at 0.25 Hz (*t*(6)  =  2.03, *p*  =  .088, $d_{z}=0.77$) and 0.50 Hz (*t*(6)  =  2.27, *p*  =  .064, $d_{z}=0.86$), these differences did not reach s*t*atistical significance. These results suggest *t*hat pupil constriction tended to be stronger when the spatial frequency components of the attentional target alternated from LSF to HSF condition than in the opposite direction, although this effect became less robust after the second alternation.

In the condition with dynamic icon images starting with HSF components (lower panels in Fig 11(b)), the slopes of pupil change after the first alternation were also calculated. Paired-samples *t*-tests revealed significant differences in pupil change slopes between the LSF-to-HSF and HSF-to-LSF conditions across all alternation frequencies. At 0.16 Hz, a highly significant difference was observed (*t*(6)  =  5.55, *p*  =  .001, $d_{z}=2.10$). Similar significant differences were observed at 0.25 Hz (*t*(6)  =  2.72, *p*  =  .035, $d_{z}=1.03$) and 0.50 Hz (*t*(6)  =  3.72, *p*  =  .010, $d_{z}=1.41$). The slopes of pupil change after the firs*t* alternation across conditions are shown in Fig 11(b).

For the second alternation, paired-samples *t*-tests were also conducted. At 0.16 Hz, the LSF-to-HSF condition induced significantly greater pupil constriction than the HSF-to-LSF condition (*t*(6)  =  5.05, *p*  =  .002, $d_{z}=1.91$), indicating a large effect size. At 0.25 Hz, the same trend was observed (*t*(6)  =  2.40, *p*  =  .054, $d_{z}=0.91$), although the difference did not reach s*t*atistical significance. At 0.50 Hz, the difference was again significant (*t*(6)  =  2.60, *p*  =  .041, $d_{z}=0.98$). Across all frequencies, the LSF-to-HSF condition generally elicited greater pupil constriction *t*han the HSF-to-LSF condition, suggesting that pupil responses remained sensitive to the direction of spatial frequency alternation. However, compared with the first alternation, the statistical evidence was weaker after the second alternation, indicating that pupil responses immediately following the first alternation provide greater potential for attention inference.

## Classification of attentional target

To classify which icon image subjects were focusing on, a classification method was used that relied on two pupil-related features independently: the absolute change in pupil size following stimulus onset and the slope of change in pupil size after the first alternation in spatial frequency. Based on the procedures described in the previous sections, the absolute pupil change was defined as the difference between the maximum and minimum pupil diameter within a 1-second time window beginning 0.5 seconds after stimulus onset. The slope of pupil change was defined as the coefficient of a linear regression fitted to the pupil change within a 1-second window beginning 0.5 seconds after the stimulus alternation. Results from Experiments 1–3 demonstrated that pupil dynamics vary systematically depending on the spatial frequency condition of the target icon. Specifically, pupil size tended to be smaller when attention was directed toward HSF images compared to LSF images. Moreover, a stronger pupil constriction was observed when the target alternated from LSF to HSF rather than from HSF to LSF.

These findings suggest that, when the magnitude of pupil change is sufficiently pronounced, it is feasible to infer the attended target by applying a threshold-based classification approach.

The optimal threshold was determined using receiver operating characteristic (ROC) analysis, with Youden’s J statistic (J=TPR−FPR) used to identify the point that best separated the two conditions [25,26]. Each pupil measure (absolute change or slope) was paired with its corresponding truth label, indicating whether the attentional target image was high spatial frequency (HSF) or low spatial frequency (LSF), and whether the pupil response reflected a transition from HSF to LSF or from LSF to HSF components. Because attending to LSF images induces greater pupil size, absolute pupil change values below the threshold suggest attention to HSF after stimulus onset, whereas values above the threshold suggest attention to LSF. Similarly, a slope value below the threshold indicates a transition from LSF to HSF, and vice versa. Thus, deviations in absolute pupil change or slope relative to the threshold provide a quantitative marker of the attentional target.

The inference rule is illustrated using the absolute pupil change before alternation and the pupil change slope within a 1-second time window after alternation as representative features. For each trial, the absolute pupil change *c* before alternation and the slope value *s* within the 1-second analysis window $W^{*}$ starting 0.5-second after alternation were computed and compared with individual thresholds $\theta^{c}$ $\theta^{s}$ to infer the attended object, independently.


$$\begin{array}{c} \hat{y}=\left\{ \begin{array}{cc} \text{LSF}, & c>\theta^{c},\\ \text{HSF}, & c<\theta^{c}. \end{array}\right. \end{array}$$ (5)

$$\begin{array}{c} \hat{y}=\left\{ \begin{array}{cc} \text{HSF}\to \text{LSF}, & s>\theta^{s},\\ \text{LSF}\to \text{HSF}, & s<\theta^{s}. \end{array}\right. \end{array}$$ (6)

To evaluate classification performance, a 3-fold cross-validation was applied separately to each subject [27]. Each subject completed three experimental sessions, and the data trials were divided into three subsets according to session number. In each iteration, two subsets (e.g., sessions 1 and 2) were used as training data to estimate the optimal threshold for classifying pupil responses (HSF to LSF vs. LSF to HSF), while the remaining subset (e.g., session 3) served as the test set. The accuracy from the test set was recorded, and this procedure was repeated until each subset had served once as the test set. The individual classification accuracy for each subject was calculated as the mean across the three tested folds. The process was repeated for every subject. Finally, the averaged classification accuracy across the subjects was calculated to obtain the overall performance. Table 1 provides a summary of classification accuracies across experiments, calculated from pupil data following the first alternation.

**Table 1:** Cross-validated classification accuracies (mean ± SE, in %) during the post-alternation interval across experiments and stimulus conditions.

Exp.	Condition	0.16	0.25	0.33	0.50
1	Static–Static	75.0 ± 15.1	–	66.0 ± 17.0	68.0 ± 8.8
2	Dynamic–Dynamic	73.6 ± 2.6	67.3 ± 1.4	–	65.5 ± 2.2
3	Dyn.(L→H) + Static	77.0 ± 1.7	71.8 ± 3.4	–	66.3 ± 1.5
	Dyn.(H→L) + Static	73.0 ± 2.9	69.0 ± 2.8	–	67.5 ± 1.9

**Experiment 1.** For the before-alternation datasets, classification performance remained at chance level (around 50%), indicating that pupil responses before alternation were insufficient to infer the attentional target. After alternation, classification accuracy reached 75.0% at 0.16 Hz, while performance at higher frequencies was lower, with 66.0% at 0.33 Hz and 68.0% at 0.50 Hz (Table 1). These results suggest that pupil change can moderately distinguish attentional targets after alternation, with relatively strong performance at low (0.16 Hz) alternation frequencies.

**Experiment 2.** As in Experiment 1, classification accuracy before alternation remained close to chance level (around 50%). After the first alternation, accuracies were 73.6% at 0.16 Hz, 67.3% at 0.25 Hz, and 65.5% at 0.50 Hz (Table 1). These findings confirm that pupil-based classification improves after alternation, with the strongest performance again observed at lower alternation frequencies, particularly 0.16 Hz.

**Experiment 3.** Consistent with the first two experiments, before-alternation classification accuracy was close to chance (around 50%). After the first alternation, classification performance improved across the combined stimulus conditions. For the condition combining *Dynamic (LSF to HSF)* with *Static (HSF to LSF)* icons, accuracies were 77.0%, 71.8%, and 66.3% at 0.16 Hz, 0.25 Hz, and 0.50 Hz, respectively. For the reverse combination, *Dynamic (HSF to LSF)* with *Static (LSF to HSF)* icons, accuracies were 73.0%, 69.0%, and 67.5% at 0.16 Hz, 0.25 Hz, and 0.50 Hz, respectively (Table 1). These results suggest that combining dynamic and static icon images enhances discriminability and that pupil-based classification remains most effective at lower alternation frequencies.

**Overall.** Across the three experiments, pupil responses consistently yielded chance-level classification before alternation but achieved accuracies of approximately 65–77% after the first alternation, with the highest performance observed in Experiment 3 at 0.16 Hz. These results confirm the feasibility of decoding attentional targets from pupil dynamics and indicate that lower alternation frequencies were descriptively associated with higher classification accuracy, while conditions containing dynamic stimuli generally showed lower variability and, in one Experiment 3 condition, the highest observed mean accuracy. Although sessions were conducted in order and subjects may have experienced fatigue or sleepiness throughout the experiments, the relatively small variability observed in Experiments 2 and 3 suggests that classification performance was more consistent across subjects than in Experiment 1. However, larger SE values were observed in Experiment 1, indicating substantial inter-subject variability, with some subjects performing near chance and others achieving perfect accuracy. This suggests that, although the mean classification accuracy in Experiment 1 was comparable to that of Experiments 2 and 3, the stability of classification with static stimuli was lower. In contrast, dynamic hybrid stimuli appeared to provide more stable performance and may offer greater potential for practical application.

## Discussion

This study investigates pupillary responses when subjects focus on one of the icon images in a hybrid stimulus. The spatial frequency components of icon images alternated between high spatial frequency (HSF) and low spatial frequency (LSF), and the icon images were dynamic or static.

In Experiment 1, both icon images in a hybrid stimulus were static, and pupil data before and after the alternation were analyzed. Before the alternation, no significant differences in pupil responses were observed between the HSF and LSF attentional target conditions under any alternation frequency. Although the 0.33 Hz condition showed a relatively larger effect size than the other frequencies, the difference did not reach statistical significance. Classification accuracy based on pupil responses before alternation remained close to chance level across all alternation frequencies. For the slope of pupil change in the after-alternation phase, significant differences were found across all conditions, along with greater classification accuracy. This is likely because the alternation of spatial frequency components required continuous updating of attentional selection and induced higher cognitive load, requiring re-engagement with new visual information. Increased cognitive load is generally associated with pupil dilation and greater attentional effort [28,29]. However, pupil constriction occurred when spatial frequency components changed from LSF to HSF, possibly reflecting the visual system’s sensitivity to changes in spatial frequency components [30]. These findings suggest that the pupil response elicited by spatial frequency alternation, rather than the absolute pupil change before alternation, provides the primary information for inferring the attended icon.

Experiments 2 and 3 aimed to enhance visual salience using dynamic images, thereby promoting stronger attentional engagement and enhancing changes in pupil responses. In Experiment 2, both icon images in a hybrid stimulus were dynamic. When comparing the pupil change slopes between the LSF-to-HSF and HSF-to-LSF alternation conditions, Experiment 2 exhibited descriptively larger differences than Experiment 1. This observation suggests that introducing dynamic stimuli may enhance the pupil response associated with spatial frequency alternation. Despite these significant group-level differences, the classification accuracy of attentional icon images based on pupil responses after the first alternation was comparable to that observed in Experiment 1. In Experiment 3, one dynamic icon image was combined with a static icon image in a hybrid stimulus, and the attentional target was either the dynamic or static icon. This manipulation likely increased visual motion contrast between the two icon images, not only in spatial frequency (HSF vs. LSF) but also in motion status (dynamic vs. static), thereby enhancing perceptual distinction and facilitating attentional allocation. As a result, the pupil dynamics elicited by alternation of spatial frequency components became more distinct and may have contributed to the accuracy of inference based on pupillary responses.

Among all conditions in Experiments 1–3, the greatest classification accuracy (77%) was observed when the hybrid stimulus consisted of dynamic and static icons with an alternation frequency of 0.16 Hz. This combination should have the strongest potential for use in a communication support system to infer attentional targets. Across experiments, there was also a trend that lower alternation frequency yielded greater classification accuracy. This may be because the pupil requires longer reaction times to respond to changes in spatial frequency components. Longer exposure to a given attentional target image may allow greater accumulation of top-down cognitive processing or sensory adaptation, amplifying pupil responses. In contrast, faster alternation may not allow sufficient time for full pupil responses or for attention to shift quickly enough.

There are at least three possible reasons why different pupil responses occur when attention is directed to images with different spatial frequency components. First, there is a difference in perceived brightness. Although all hybrid images were processed to have the same overall brightness, HSF icons may be perceived as ’brighter’ due to greater edge density and contrast, potentially leading to pupil constriction [31], whereas LSF icons may be perceived as ’darker’ due to smoother edges and lower contrast [32]. Second, although the physical distance of the icon images was the same, subjects reported that HSF components appeared ’closer’, while LSF components seemed to be placed behind. A previous study has demonstrated that objects perceived or imaged as nearer induce pupil constriction [33], reflecting the pupillary near response (PNR) observed in natural environment [34]. In the real world, focusing on nearer objects induces pupil constriction to increase depth of focus, while focusing on distant objects induces pupil dilation [30]. Therefore, the pupil change in response to icons with different spatial frequencies is also consistent with the above previous findings showing that perceived or imagined distance can modulate pupil size. Third, there may be a difference in cognitive load. HSF images are more salient and easier to focus on due to their clearer edges. In contrast, LSF images require relatively greater mental effort to distinguish and to focus on, especially when ignoring the overlaid HSF image. The increased cognitive load may result in larger pupil size when attending to LSF images [28].

Building on these findings, it is possible to propose a communication support system that uses these pupil response patterns to infer attentional targets. By designing image sets that address patients’ core needs, the system enables users to generate specific commands through two to three rounds of binary selection. For example, by attending to icon images representing “controller,” “light,” and “on” in three hybrid stimuli sequentially, the system can interpret and execute the command “turn on the light.” When connected to external devices, such a system offers individuals with conditions such as CLIS in ALS an expanded means of communication, enabling them to communicate beyond simple yes/no responses and regain a sense of control in their environment. Moreover, the trial duration in this study was intentionally designed to examine whether subjects would show improved performance after repeated alternations within a trial (e.g., after the second alternation), as they became more familiar with the task and target stimuli. However, our results indicate that the classification based on the first alternation alone already achieves sufficiently good performance. This suggests that multiple alternations within a single trial are not necessary for reliable inference. Therefore, in practical applications, the trial can be redesigned to include only a single alternation, which would substantially reduce the trial duration and improve usability for communication purposes. Moreover, individual differences in pupillary dynamics should be considered for further practical use. In Experiments 2 and 3, pupil response latency varied substantially across subjects, ranging from approximately 0.78 s for Subject 1 to over 1.1 s for Subject 3, while remaining relatively stable across trials with each subject. These results suggest that the timing of the strongest pupil response may differ across subjects. Classification accuracy based on the fixed time window also varied across subjects, which may partly reflect these temporal differences in pupil dynamics. See the supplementary table for the detailed individual latency and accuracy results. Therefore, applying a fixed temporal window to all users may not be optimal. Future studies may benefit from using sliding-window approaches to identify subject-specific temporal windows that improve decoding accuracy.

In conclusion, our results demonstrate that pupillary responses can serve as a reliable, non-invasive index of visual attention, particularly when attentional targets are distinguished by multiple visual features such as spatial frequency and motion. Furthermore, these results may contribute to the development of attention-based communication support systems for individuals with severe motor impairments. Such systems would not require eye movement signals but instead rely on pupil responses, one of the fundamental vital signs in humans.
